# Target versus non-target lesions in determining disease progression: analysis of 545 patients

**DOI:** 10.1186/1470-7330-15-S1-S8

**Published:** 2015-10-02

**Authors:** S Raskin, E Klang, M Amitai

**Affiliations:** 1 Sheba Medical Center, Ramat Gan, Israel

## Aim

RECIST and other methodologies emphasise tracking the diameters of target lesions (TLs) for the determination of progressive disease (PD) in randomised clinical trials. However, RECIST 1.1 also allows for the use of non TL lesions (NTL) to determine PD. We sought to determine whether the final assessment of PD was more likely to be determined by the set of target lesions with PD (TL-PD) or by the set of non-target lesions with PD (NTL-PD).

## Methods

We evaluated the formal RECIST evaluations for consecutive patient enrollments in randomised clinical trials at our institution from 2012 to 2014. Data were grouped as TL-PD or NTL-PD, and the groups were compared. PD was further divided as to whether lesions were new or demonstrated unequivocal progression.

## Results

Of 545 cases, 341 received a final assessment of PD. Of these, TL-PD analysis by itself accounted for 180 (53%) patients, and NTL-PD analysis itself accounted for 287 (84%). In the NTL-PD group, 142 (42%) had either Partial Response (PR) or Stable Disease (SD) according to TL analysis. Among all instances of NTL-PD, new, measurable disease was the most common determinant of PD, as seen in 210 (73%) instances.

## Conclusion

In this series of patients enrolled in clinical trials, NTL analysis was more likely to result in a determination of PD than tracking TL diameters, and the development of new, measurable disease was the most common determinant of PD. These findings may have relevance for the design of new methodologies for the assessment of tumour response.

